# Fungi associated with mesophotic macroalgae from the ‘Au‘au Channel, west Maui are differentiated by host and overlap terrestrial communities

**DOI:** 10.7717/peerj.3532

**Published:** 2017-07-11

**Authors:** Benjamin J. Wainwright, Geoffrey L. Zahn, Heather L. Spalding, Alison R. Sherwood, Celia M. Smith, Anthony S. Amend

**Affiliations:** Department of Botany, University of Hawaii at Manoa, Honolulu, HI, United States of America

**Keywords:** Connectivity, ITS, Marine, Hawaii, Biodiversity, Mesophotic coral ecosytems, Fungi

## Abstract

Mesophotic coral ecosystems are an almost entirely unexplored and undocumented environment that likely contains vast reservoirs of undescribed biodiversity. Twenty-four macroalgae samples, representing four genera, were collected from a Hawaiian mesophotic reef at water depths between 65 and 86 m in the ‘Au‘au Channel, Maui, Hawai‘i. Algal tissues were surveyed for the presence and diversity of fungi by sequencing the ITS1 gene using Illumina technology. Fungi from these algae were then compared to previous fungal surveys conducted in Hawaiian terrestrial ecosystems. Twenty-seven percent of the OTUs present on the mesophotic coral ecosystem samples were shared between the marine and terrestrial environment. Subsequent analyses indicated that host species of algae significantly differentiate fungal community composition. This work demonstrates yet another understudied habitat with a moderate diversity of fungi that should be considered when estimating global fungal diversity.

## Introduction

Mesophotic coral ecosytems (MCEs) remain an almost entirely unexplored and undocumented environment that likely contains vast reservoirs of undescribed biodiversity (Pyle et al., 2016; Baker, Puglise & Harris, 2016). Within the Hawaiian Archipelago, these low-light reefs are found at depths between 40 and 130 m whereas globally MCEs have been reported in depths extending to more than 150 m (Kahng & Kelley, 2007; Hinderstein et al., 2010; Pyle et al., 2016). MCEs extend to depths that are unsuitable for the application of traditional open circuit SCUBA techniques and are generally too shallow for frequent exploration by human-piloted submersible vehicles (Pyle, 1996; Pyle, Earle & Greene, 2008). Consequently, little is known of the biodiversity, community composition and importance of these MCEs. The application of mixed gas, closed circuit (Rebreather) diving techniques along with Remotely Operated Vehicles (ROVs) and Autonomous Underwater Vehicles (AUVs) are now making exploration of these understudied areas of the ocean possible (Pyle, Earle & Greene, 2008; Bridge et al., 2011). Nevertheless, these mesophotic ecosystems remain far less studied than reefs at shallower depths. For example, a Web of Science Core Collection search for “Mesophotic” returned 181 results, while a search for “Coral Reefs” returned 22,957 results, demonstrating the comparatively limited research in MCEs (search performed 18 Nov 2016).

Fungi have been documented in almost all of the habitats found on Earth, although marine fungi are less studied in comparison to their terrestrial counterparts (Blackwell, 2011; Richards et al., 2012; Peay, Kennedy & Talbot, 2016). Recent research shows that marine fungi contain a high diversity of putatively novel taxa (Comeau et al., 2016; Ishino et al., 2016; Picard, 2017), some of which may have medical applications (Hasan et al., 2015; Zin et al., 2016). Perhaps the best-described communities of reef-associated fungi are those growing on or in corals. The association of coral with marine fungi was first documented in the mid 1800’s (Kölliker, 1859). More recently, researchers have reported finding fungi in deep and shallow water corals (Freiwald & Reitner, 1997; Bentis, Kaufman & Golubic, 2000; Amend, Barshis & Oliver, 2012; Sweet & Séré, 2016). Similar observations of fungi have been noted in marine sponges (Kobayashi, 2016; Ding et al., 2016), sessile invertebrates (Yarden, 2014) and marine macroalgae (Kohlmeyer & Demoulin, 1981; Parsons & Fenwick, 2012). Olson & Kellog (2010) reported that there were no studies describing algal associated fungal communities from MCEs. To the best of our knowledge, this is the first documented evidence confirming algal-fugal associations on MCEs.

MCEs experience exceptionally low irradiances, at water depths of 34 m and 90 m, the quantity of photosynthetically Active Radiation (PAR) is reduced to 10% and 1% of surface irradiance respectively (Pyle et al., 2016). In terms of percent cover Hawaiian MCEs are dominated by large areal stands of several species of green, brown or red macroalgae, some in such density that they are called meadows (Pyle et al., 2016). Similar observations have been made on the Pulley Ridge and the Puerto Rico insular shelf MCEs (NOAA Ocean Explorer Mission Log Aug 22, 2013; Baker, Puglise & Harris, 2016). The exact nature of the algal and fungal interactions on MCEs presently remains unknown. Previous work by Inderbitzen et al. (2004) and Küpper et al. (2006) has shown that fungi can be pathogens of marine macroalgae. Conversely, it is not unreasonable to suggest that the fungi found on and in the algal samples collected in this study could be symbionts playing an important role in host chemistry or fitness (Kohlmeyer & Demoulin, 1981; Kohlmeyer & Volkmann-Kohlmeyer, 2003).

Because we are interested in marine fungal contributions to global fungal diversity, our study focuses on determining the extent to which host identity and spatiotemporal variables correlate with fungal community composition at small spatial scales. Further, we determined the amount of overlap with nearby, terrestrial-plant associated fungi to examine connectivity with ecologically divergent habitats. We hope that this work will facilitate additional discussion on the role and importance of algal and fungal associations on MCEs and other macroalgal habitats.

## Methods

**Figure 1:**
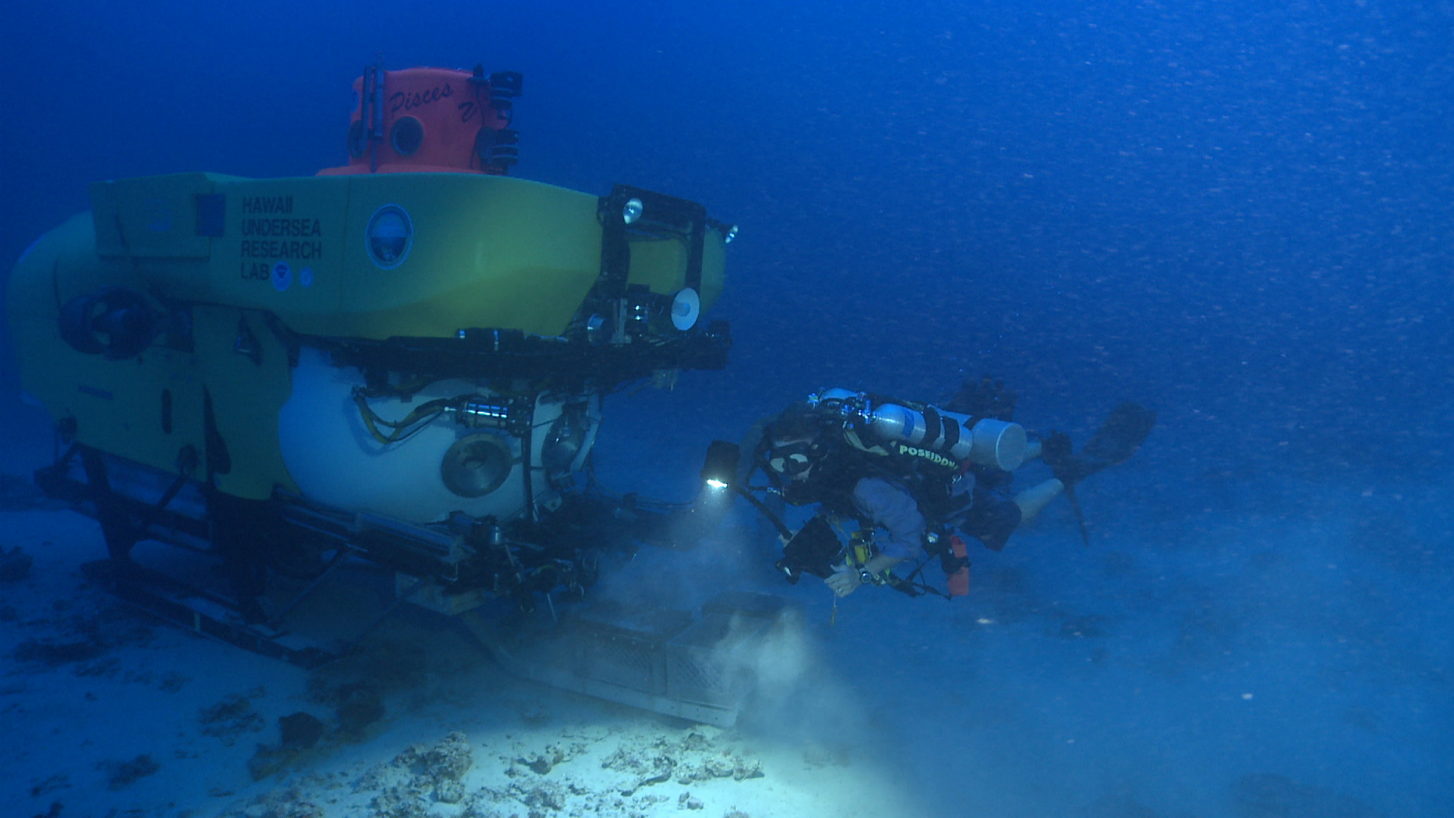
Picture is taken at ∼90 m depth and shows a rebreather diver with the submersible *Pisces* V working together collecting corals and macroalgae in the ‘Au‘au Channel (Image credit: Robert K. Whitton).

**Figure 2:**
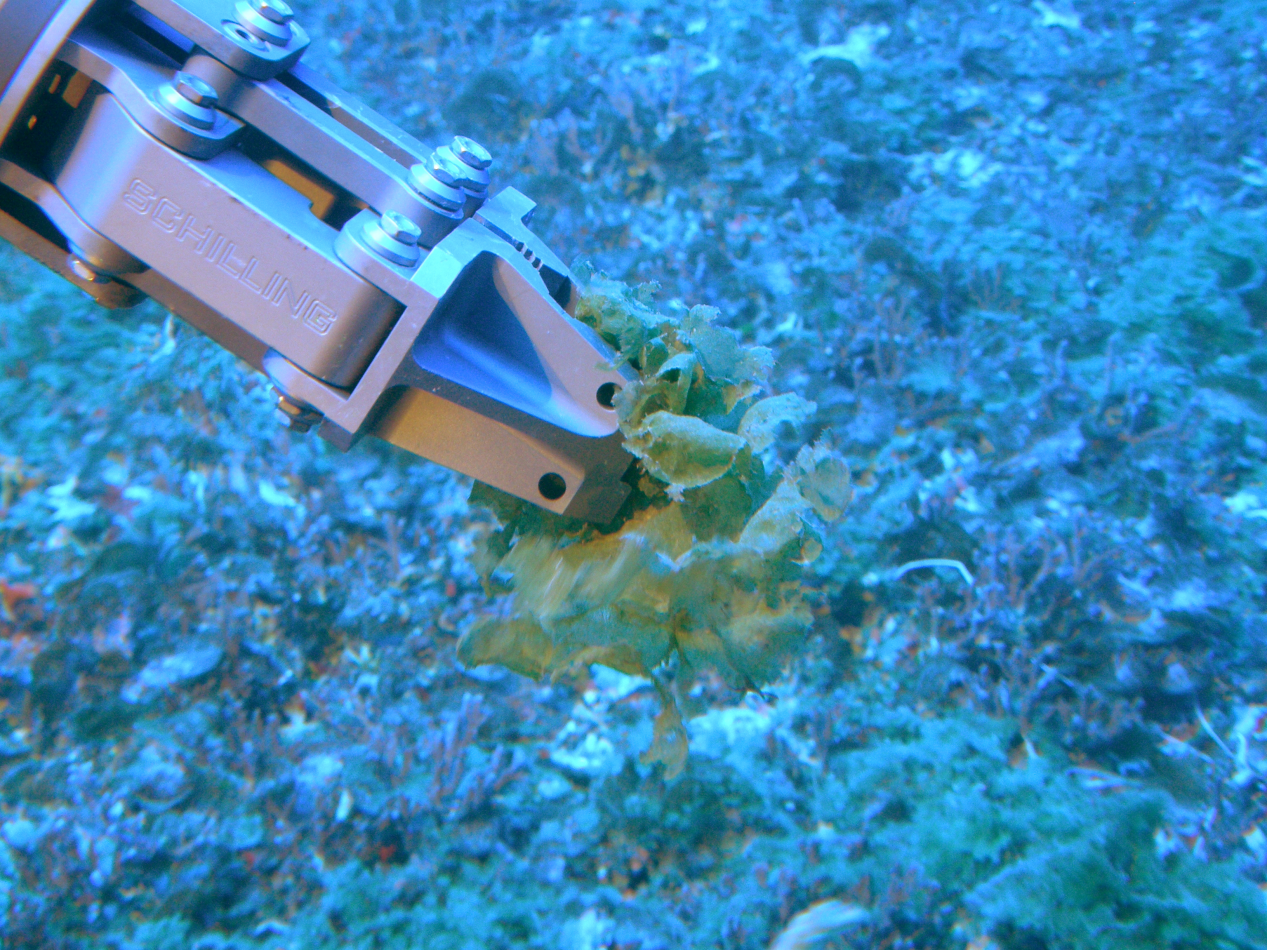
Manipulator arm of the *Pisces* V submersible collecting *Microdictyon umbilicatum* (Image credit: Hawaii Undersea Research Lab).

**Figure 3:**
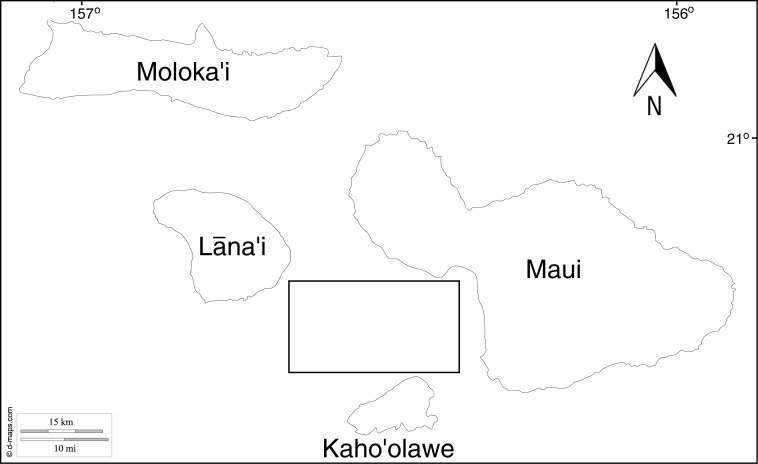
Map showing the location of sampling sites, box represents the approximate location all samples were collected from within the ‘Au‘au Channel. See Table 1 for GPS co-ordinates of collection sites. Map downloaded from http://www.d-maps.com.

Mesophotic samples of four ecologically dominant algae from three evolutionary clades (Chlorophyta, Rhodophyta and Ochrophyta; Table 1) were collected by the Hawaii Undersea Research Laboratory (HURL) using the collector arm on the *Pisces V* submersible (Figs. 1 and 2) at a range of depths (65 m and 86 m) between 18 Feb 2011 and 3 March 2011 from the ‘Au‘au Channel, west Maui (Fig. 3 & Table 1). Samples were initially stored on dry ice and transferred to a land based −80°C freezer for long-term storage. See Supplemental Information 2 (Mesophotic Macroalgae Descriptions) for examples of collected tissue and detailed descriptions of each species.

**Table 1:** Details of species, collection depth, GPS coordinates of collection location and Bishop museum accession number where available.

Species	Phylum	Sample ID	Depth (m) collected	Latitude	Longitude	Bishop museum accession number
*Distromium sp.*	Ochrophyta	B2	86	20 46.813	−156 40.813	–
*Distromium sp.*	Ochrophyta	B3	86	20 46.813	−156 40.813	–
*Distromium sp.*	Ochrophyta	B6	86	20 46.813	−156 40.813	–
*Distromium sp.*	Ochrophyta	B12	67	20 48.638	−156 43.009	–
*Distromium sp.*	Ochrophyta	B13	67	20 48.648	−156 42.983	BISH 767544^a^
*Distromium sp.*	Ochrophyta	B15	67	20 48.652	−156 42.982	BISH 767545^a^
*Distromium sp.*	Ochrophyta	B16	66	20 48.652	−156 42.982	–
*Distromium sp.*	Ochrophyta	B17	79	20 48.681	−156 42.862	–
*Distromium sp.*	Ochrophyta	B20	73	20 48.726	−156 42.904	–
*Distromium sp.*	Ochrophyta	B22	75	20 48.692	−156 42.857	–
*Halimeda distorta*	Chlorophyta	H1	86	20 46.813	−156 40.813	BISH 767546^a^
*Halimeda distorta*	Chlorophyta	H4	86	20 46.813	−156 40.813	BISH 767547^a^
*Halimeda distorta*	Chlorophyta	H5	86	20 46.813	−156 40.813	BISH 767548^a^
*Halimeda distorta*	Chlorophyta	H9	66	20 48.652	−156 42.982	BISH 767549^a^
*Halimeda distorta*	Chlorophyta	H14	85	20 46.812	−156 40.429	–
*Halimeda distorta*	Chlorophyta	H17	85	20 46.812	−156 40.429	–
*Microdicyton umbilicatum*	Chlorophyta	Mi1	67	20 48.638	−156 43.009	BISH 767552^a^
*Microdicyton umbilicatum*	Chlorophyta	Mi3	67	20 48.648	−156 42.983	–
*Microdicyton umbilicatum*	Chlorophyta	Mi4	67	20 48.652	−156 42.982	–
*Microdicyton umbilicatum*	Chlorophyta	Mi9	65	20 48.652	−156 42.982	–
*Microdicyton umbilicatum*	Chlorophyta	Mi10	73	20 48.726	−156 42.904	–
*Microdicyton umbilicatum*	Chlorophyta	Mi11	78	20 48.815	−156 42.860	–
*Halymenia sp.*	Rhodophyta	R1	86	20 46.813	−156 40.813	BISH 767550^a^
*Halymenia sp.*	Rhodophyta	R2	86	20 46.813	−156 40.813	BISH 767551^a^

**Notes.**

a Herbarium Pacificum (BISH) collection.

A section of the blade from each species weighing approximately 25 mg was placed in DNA extraction buffer solution (410 µl PD1, 40 µl PSS, 50 µl PD2 and 3 µl RNase A, MoBio, Carlsbad, CA) and disrupted in an MP Biomedicals Lysing matrix A 2 ml tissue tube (MP Biomedicals; Solon, OH, USA) using a Biospec Mini-Beadbeater-24 (Biospec product; Bartlesville, OK, USA) for 2 min at 2,000 oscillations per minute. All tissue, across all specimens, was taken from an area of the blade that had no observable epiphytes and all tissue was collected from an area of the blade that was approximately the same age (i.e., samples were not collected from the tip in one and from the base in others). Following disruption, DNA was extracted with a MoBio PowerPlant® Pro-htp 96 well DNA isolation kit following the manufacturer’s instructions. Because of the difficulties and high costs associated with the collection of these samples only 25 mg of each sample was available for molecular analysis, while the remainder was used for other physio-chemical studies.

Fungal DNA amplification of the ITS1 region was performed using the ITS1F primer (CTTGGTCATTTAGAGGAAGTAA; Gardes & Bruns, 1993) and the ITS2 primer (GCTGCGTTCTTCATCGATGC; White et al., 1990). Forward and reverse primers were modified to include Illumina adaptors, a linker and a unique barcode (see Smith & Peay, 2014 for additional details including custom sequencing primers). Each reaction was performed in a total volume of 25 µl, containing 9 µl of template DNA diluted 1:5, with final concentrations of 0.25 U of KAPA 3G Enzyme (Kapa Biosystems, Inc., Wilmington, MA, USA), 0.3 µM of each primer, 1.5 mg/mL of BSA and KAPA Plant PCR Buffer to a final concentration of 1×. PCR cycling protocol was 95°C for 3 min, followed by 35 cycles of 95°C for 20 s, 53°C for 15 s, 72°C for 20 s with a final extension at 72°C for 60 s. Negative controls were included to identify any possible contamination issues as per Nguyen et al. (2014).

PCR products were visualized on a 1% SB buffer agarose gel and cleaned with AMpure beads. Normalization of PCR products was performed with just-a-plate™ 96 PCR purification and normalization kit (Charm Biotech San Diego, CA, USA). Cleaned and normalized PCR products were quantified using a Qubit® 2.0 Fluorometer following the hs-DS-DNA protocol (Invitrogen; Carlsbad, CA, USA), pooled into equimolar amounts and submitted for sequencing on the Illumina MiSeq platform (600 cycles, V3 chemistry, 300 bp paired end reads) at the Hawai‘i Institute of Marine Biology sequencing core facility with two other unrelated libraries.

Fungal foliar epiphyte (FFE) samples were collected by swabbing leaf surfaces with sterile swabs within the Wai’anae Range, O’ahu, (see O’Rorke et al., 2015 for details). DNA was then extracted from the swabs using MoBio PowerSoil kits following standard protocol. DNA amplification was performed following the same protocol detailed for mesophotic algae. See Table S1 for additional collection details.

### OTU picking and taxonomic assignments

Bi-directional raw reads were de-multiplexed using Illumina software and assembled using PEAR (Zhang et al., 2014). Successfully assembled reads were quality filtered such that all reads with at least one base score lower than 25 were removed using the fastx_toolkit (http://hannonlab.cshl.edu/fastx_toolkit/). Quality filtered reads were checked for potential chimeric sequences in VSearch (Rognes et al., 2016) against a fungal ITS1 chimera database (Nilsson et al., 2015), and remaining singleton sequences were removed. The resulting reads were used for “open-reference” OTU picking within QIIME v1.9.1 (Caporaso et al., 2010) using the UNITE ITS1 fungal database (downloaded 2016-10-31) to which several marine metazoan outgroups were added (see Supplemental Information 3). Default settings were used throughout, with the exception that taxonomy assignment utilized the BLAST method.

The UNITE database is a meticulously curated database widely used to assign taxonomy to fungal ITS reads, and is easily deployed in common analysis pipelines such as QIIME. For this reason, among others, it is commonly used to assign fungal taxonomy to environmental ITS sequences (Raja et al., 2017; Siddique & Unterseher, 2016). The database, however, lacks representative sequences with marine origins, so we question its current utility for marine fungal studies. In fact many sequences identified to low taxonomic ranks (e.g., genus, species) of fungi using UNITE were more similar to marine metazoan sequences when compared to the NCBI nucleotide database. Because of potentially erroneous assignments of marine organisms to UNITE fungal taxonomy lineages, OTU taxonomy was evaluated as the top BLAST match to a representative OTU sequence against the NCBI nucleotide database with a minimum *e*-value of 1 × 10^−15^. Any OTU for which the top BLAST hit was not fungal was removed. All subsequent operations on and analyses of the OTU occurrence table were performed in R, version 3.3.1.

The resulting OTU table was filtered to control for barcode bleed over (Esling, Lejzerowicz & Pawlowski, 2015) by removing OTUs from samples when present at less than 0.1% of the maximum OTU abundance found elsewhere. Further, to achieve a conservative OTU richness estimate and eliminate sequencing artifacts, OTUs represented by fewer than five reads were removed (Laroche et al., 2017). The OTU table was then subsampled to 4,000 reads per sample using the vegan package (Okansen et al., 2016).

To compare the overlap between plant-associated fungi in marine and terrestrial environments, we also combined the MCEs sequences with those from a study taken from angiosperm fungal foliar epiphytes (FFE) on the island of O‘ahu. MCE OTUs were compared to FFE OTUs via uclust (Edgar, 2010), in which MCE OTUs were re-picked by clustering representative sequences with the uclust_ref algorithm in QIIME using FFE representative sequences as a reference database at 97% similarity, and with suppression of new OTU clusters. The OTUs that successfully clustered with FFE sequences were accepted as shared between the two ecosystems. By comparing the MCEs samples with a more well-studied but nearby ecosystem, we can estimate the novelty of the communities in this previously-unstudied MCE.

All sequences used in this study have been deposited in the Sequence Read Archive database (Accession Numbers: PRJNA355011 for FFE; PRJNA355018 for MCEs).

### Statistics and community analyses

To determine how algal species, reef depth, and sampling dive (spatio-temporal factor) influence fungal communities, model-based analyses of multivariate fungal abundance were performed with anova.manyglm function in the mvabund package (Wang et al., 2012). Non-metric Multi-Dimensional Scaling (NMDS) analysis was performed with the metaMDS function in the vegan package using a Bray Curtis community distance metric (Fig. S1).

## Results

### Bioinformatics results

Sequencing resulted in 279,431 raw forward and reverse reads, of which 255,830 were successfully assembled. After quality filtering and removal of chimeras and singletons, the remaining 209,508 reads were used to cluster OTUs. Sequences were clustered into 147 different fungal OTUs. One sample, “B20”, only yielded three reads and was excluded from subsequent analyses (Table S2).

### Overview of fungal OTUs and “species” found in MCEs

**Figure 4:**
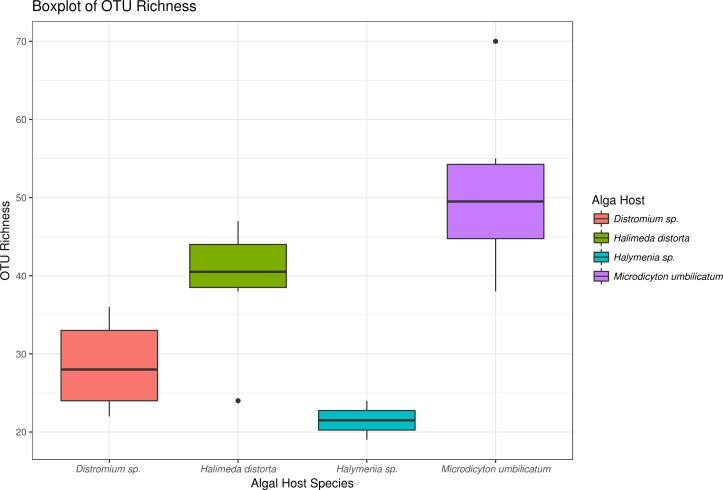
Boxplot showing richness of OTUs from each algal host.

Of the 147 fungal OTUs found in the MCE algal biome, 39 were also found among the foliar epiphytes (Table S3). The most common OTUs from the MCEs samples were dominated by *Alternaria*, an undetermined uncultured fungus, *Cladosporium,* and *Rhodotorula* species (Table S4). OTUs were predominantly undescribed (top blast hit = uncultured fungus), but described hits were dominated by *Cladosporium* spp. Although 39 OTUs were shared between the two ecosystems, they displayed different patterns of abundance (Fig. 4; Tables S5 & S6). 17 OTUs were found in all samples collected from MCEs, of these only one was shared between MCE and FFE (Table S7).

### Effect of algal host on OTU diversity and identity

Host algal identity played a significant role in determining fungal community composition (MANOVA: Wald (3, 19) = 14.14; *P* = 0.033) with the alga *Microdictyon umbilicatum* showing significant variation in fungal community structure compared to other algal hosts (Table S8). *M. umbilicatum* was also host to a greater richness of fungal OTUs than other algae (Fig. 4). Adjusted univariate test statistics were calculated for each OTU within the mvabund package (Wang et al., 2012) for a generalized linear model and revealed no significant correlations between any OTU and algal species or sampling dive. Other predictive variables (Spatio-temporal, light level, temperature, depth) did not correlate with observed fungal community structure.

## Discussion

For every hour spent exploring the depths where MCEs are found, seven new species of fish are described and conservative extrapolations suggest that more than 2,000 species of reef-associated fish have yet to be described from these habitats (Pyle, 2000). Similar or even greater numbers of other taxa likely remain undescribed on MCEs, especially fungi and other microbes. Consequently, work in these unexplored environments is important for accurate assessments of fungal and microbial diversity as well as assessments of total global biodiversity.

Earlier work examining global drivers of host-independent marine fungal diversity (Tisthammer, Cobian & Amend, 2015) found that habitat and abiotic environmental factors were the greatest determinants of fungal community composition. We show that host-association adds a layer of complexity to fungal community structure in marine habitats. As found with terrestrial plants (Kembel & Mueller, 2014; O’Rorke et al., 2015), host species in the MCEs weakly predicted fungal community composition. With the data set at hand, we are unable to explain why these differences in fungal community exist, and many questions about the ecological role, interactions, physiology and compositional stability of these communities remain. Further work will investigate whether the observed differences in fungal community structure are a result of host traits, relatedness, or potential co-evolution.

Despite the novelty of the study environment, fungal composition showed surprising similarity with terrestrial habitats. At water depths between 65 and 86 m, where the algal samples used in this work were collected, solar irradiance, temperature fluctuations, pressure and nutrient availably differ markedly from terrestrial environments. The overlap in occurrence of fungal OTUs from Hawaiian mesophotic algae and foliar epiphytes (39 out of 147 OTUs (27%)) reveals a surprisingly high level of presumed connectivity between distinct environments containing seemingly few opportunities for dispersal. We are unable to eliminate the possibility that some of the OTUs shared between ecosystems may be derived from spores that enter the water column and passively adhere to the algal surface, consequently they may not be functionally active having no detrimental or beneficial properties to the algae they are found on. However, we can rule out contamination as an explanation because negative controls revealed no detectable environmental DNA in our reagents or lab environment.

**Figure 5:**
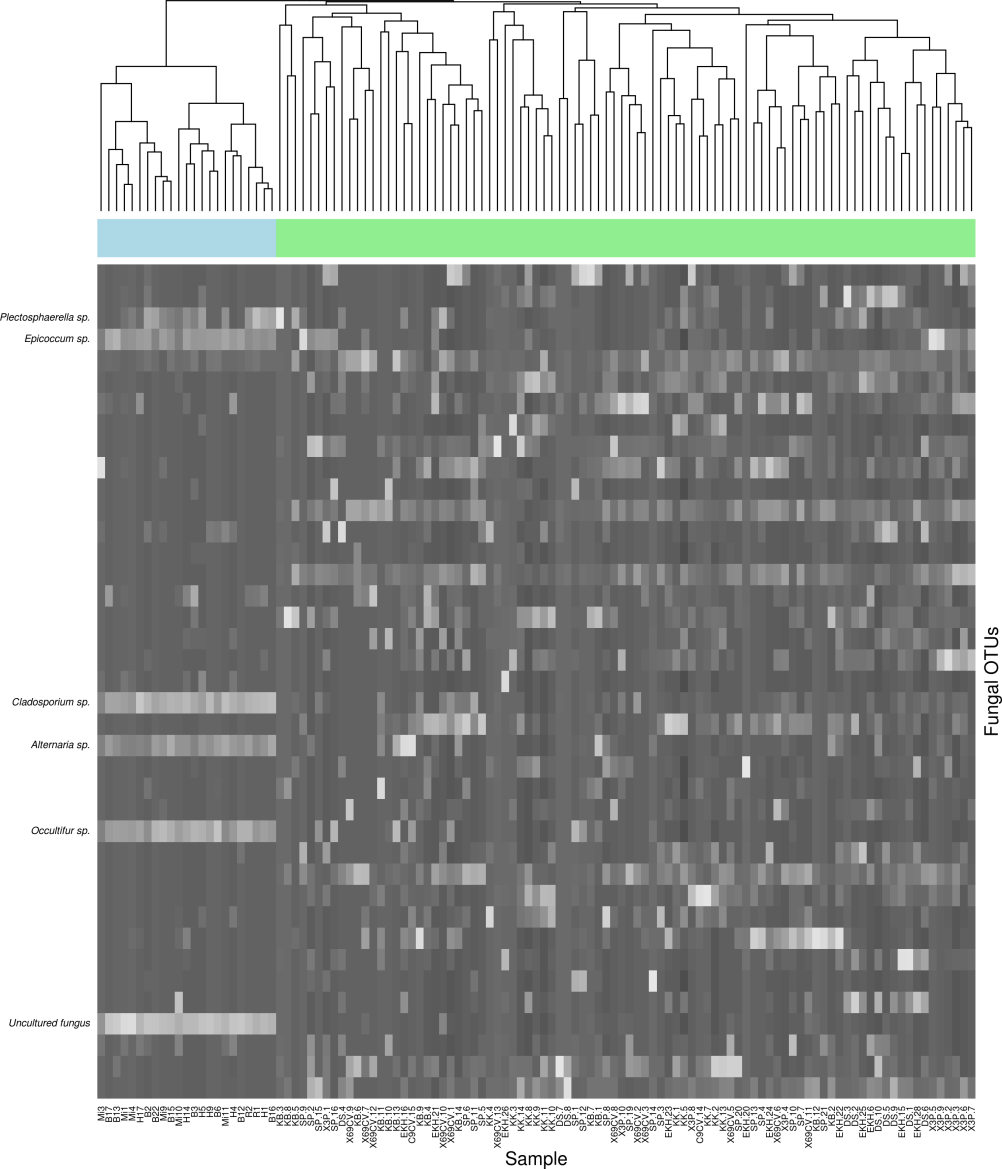
Heatmap showing relative abundance of shared OTUs found in mesophotic aglae and terrestrial sites. Only the 39 OTUs found in both the MCE and FFE ecosystems are shown (rows). Lighter color indicates greater relative abundance. The cladogram is based on Bray–Curtis dissimilarity. Samples (columns) shown in blue are from MCE and samples shown in green are from FFE. Six OTUs of interest (those shared OTUs that are reliably more abundant in MCE samples) are labeled with their top BLAST hit taxonomic assignments.

Additionally, the marine fungal community is most similar to itself when compared to the FFE community (Fig. 5), the six-labeled OTUs of interest (Fig. 5) show the greatest abundance in MCEs, suggesting that these OTUs may have a preference for the marine environment and their presence on MCEs may not be solely the result of passive spore transport or incidental adhesion of spores to the algal surface. Interestingly, other studies (Amend, Barshis & Oliver, 2012; Amend, 2014; Richards et al., 2012) have noted a preponderance of putatively amphibious fungi, although mechanisms for adaptation or acclimation, or rates of gene flow between terrestrial and marine population are not known. The selective pressures between the terrestrial and marine environment are likely to be very different for any fungi that can make the transition from one environment to another. These differences could facilitate genetic differentiation and given enough time, speciation could result, which in turn contributes to global fungal diversity. Using molecular markers with greater discriminative power may indicate population structure indicative of incipient speciation.

Very few of the papers estimating global fungal diversity incorporate marine data, and the fact that 27% of the fungal OTUs found in this MCE are also encountered in a terrestrial environment >1,200 m in elevation on the island of O‘ahu may serve as an indicator for the potential discovery of novel species in the mesophotic zone. It is possible that these novel species contain or produce compounds and secondary metabolites that are valuable in the treatment of disease and other medical ailments (e.g., cancer, statin production) or have industrial uses as enzymes (Østergaard & Olsen, 2010; Beattie & Ulrich, 2011). Some fungi may even have utility in the bioremediation of industrial waste and oil spills (Atalla et al., 2010; Garzoli et al., 2015). Despite sampling only a small portion of the entire algal structure (25 mg of the blade) we still found predominantly undescribed OTUs suggesting that many more novel OTUs remain to be discovered on parts of the algae not examined (e.g., reproductive structures) and the potential for discovery of new compounds beneficial to society is high.

Although our inferential power in this study is limited by low replication and the haphazard nature of our collections, this work indicates that there are algae-associated fungi in these habitats. Seventeen OTUs were found in all samples collected from MCEs, only one of these OTUs was shared with terrestrial FFE samples, suggesting a degree of fungal and host specialization that could be unique to MCEs. Further research into the chemistry, physiology and dispersal mechanisms is needed to gauge the ecological role fungi may be playing, their importance in nutrient cycling and potential role in facilitating host survival in low light and nutrient environments.

While many questions remain regarding the structure, functioning and community dynamics that shape MCEs; even less is known of the interactions and role that fungi could be playing on these reefs. If these MCEs are acting as “life boats” and “species refuges” for imperiled shallow water coral reef habitats as is hypothesized (Baker, Puglise & Harris, 2016; Vaz et al., 2016) it will become increasingly important that we understand the composition and functioning of this ecosystem along with the possible roles fungi play in sustaining these unique and understudied marine habitats.

##  Supplemental Information

10.7717/peerj.3532/supp-1 Figure S1 SI Figure 1- NMDS plot of fungal community similarity, colored by algal host species

10.7717/peerj.3532/supp-2 Supplemental Information 1 Supplemental Tables 1–8

10.7717/peerj.3532/supp-3 Supplemental Information 2 Details of mesophotic macroalgae

10.7717/peerj.3532/supp-4 Supplemental Information 3 OTU Picking workflow

## Additional Information and Declarations

### DNA Deposition

The following information was supplied regarding the deposition of DNA sequences: All sequences used in this study have been deposited in the Sequence Read Archive database (Accession Numbers: PRJNA355011 for FFE; PRJNA355018 for MCEs).

### Data Availability

The following information was supplied regarding data availability: The raw data has been supplied as Supplementary File.
