# Supplementary figures and images for: Fungi associated with mesophotic macroalgae from the ‘Au‘au Channel, west Maui are differentiated by host and overlap terrestrial communities
Source: PeerJ. 2017 Jul 11;5:e3532. doi: 10.7717/peerj.3532 (PMC5508810; doi:10.7717/peerj.3532)

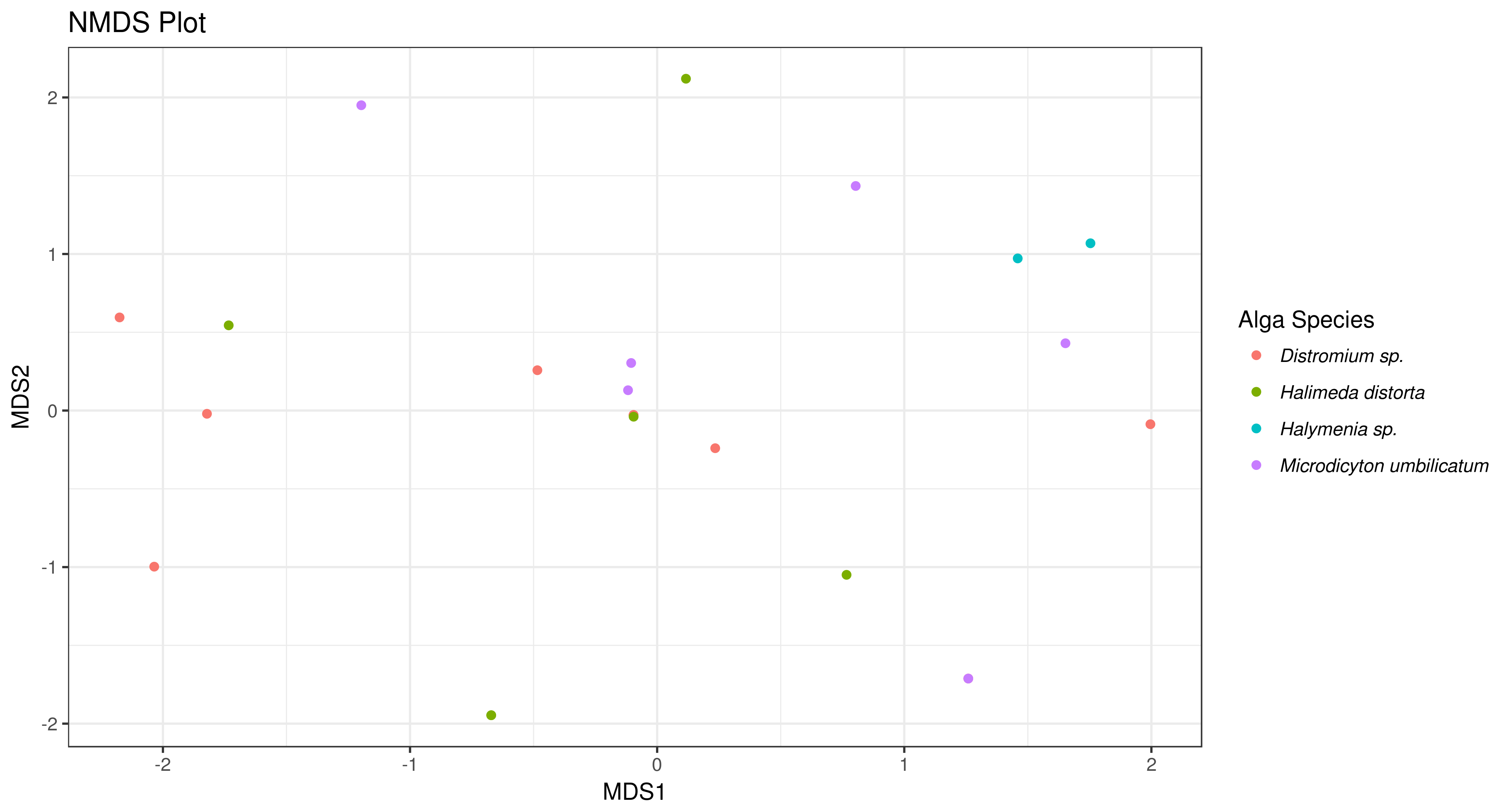

Supplement: Figure S1 [file peerj-05-3532-s001.png]
