# Supplementary material for: Fungi associated with mesophotic macroalgae from the ‘Au‘au Channel, west Maui are differentiated by host and overlap terrestrial communities
Source: PeerJ. 2017 Jul 11;5:e3532. doi: 10.7717/peerj.3532 (PMC5508810; doi:10.7717/peerj.3532)
Supplement: Supplemental Information 2 [file peerj-05-3532-s003.docx]

Species descriptions and corresponding pictures

***Halimeda distorta* (Yamada) Hillis-Colinvaux**

*Halimeda distorta* is a calcified green alga (Chlorophyta) belonging to the siphonous order Bryopsidales. Its thallus is composed of flattened, calcified segments that are attached to the substrate by several diffuse and inconspicuous patches of rhizoids. It is typically found on hard substrata, either as small mounds (~1 m^2^) or as larger (~5 – 100 m^2^) beds. Spalding (2012) observed *H. distorta* stands cascading down from the edges of mesophotic coral (*Leptoseris* spp.) reefs in Hawai‘i. Small individuals were also observed scattered from 40 - 138 m depths in low abundance and mixed with other macroalgal assemblages. Encrusting invertebrates, including sponges, tunicates, and bryozoans, and red macroalgae (Rhodophyta) were found entangled in the larger *H. distorta* mounds. Given the high abundance of epiphytes and encrusting organisms, these plants appeared to be long-lived and were likely several months, if not years, in age.


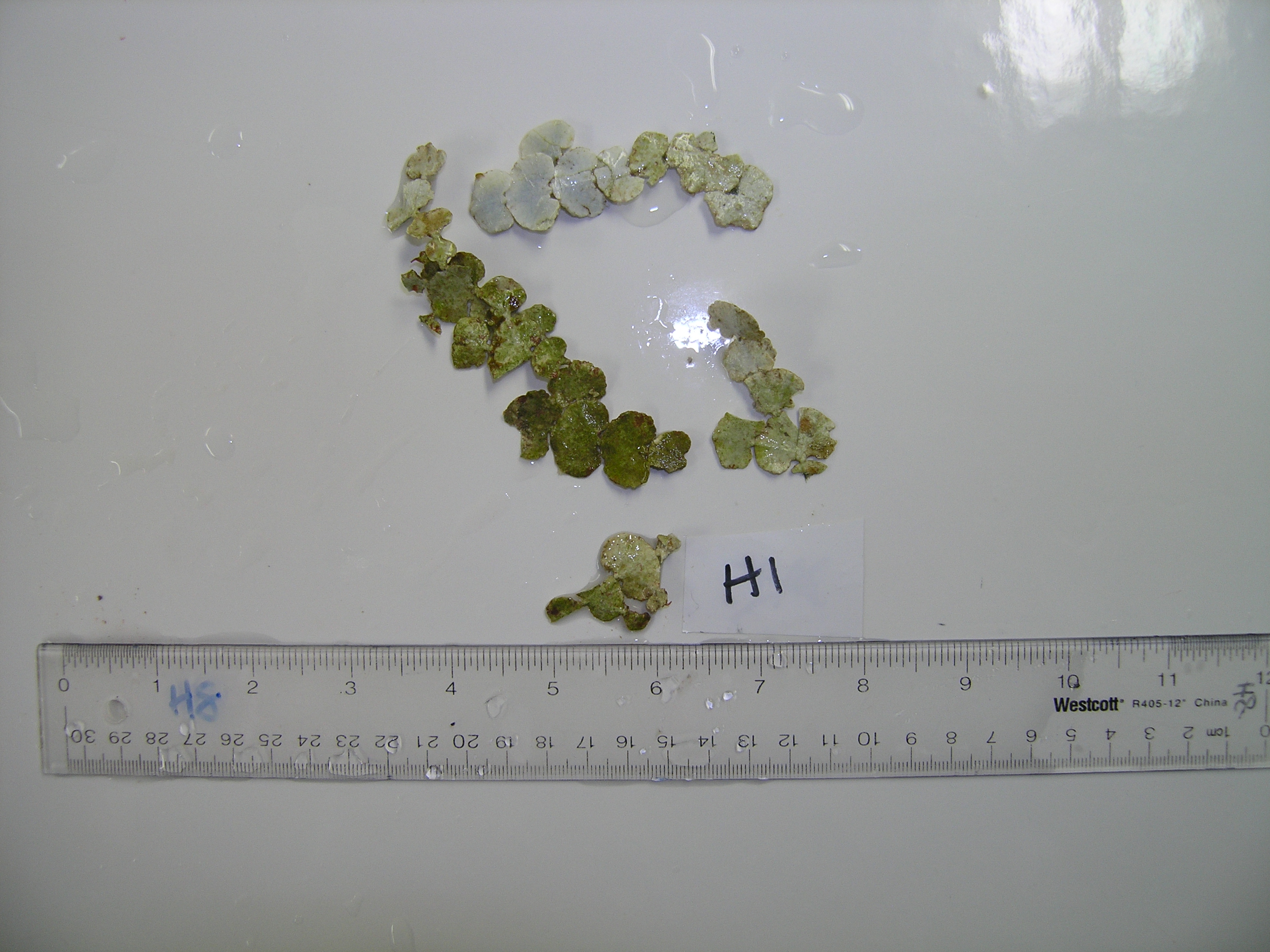


***Microdictyon umbilicatum* (Velley) Zanardini**

*Microdictyon umbilicatum* is a green alga (Chlorophyta) belonging to the order Cladophorales. It forms a flattened frond composed of a fine, delicate, mesh-like structure, and often forms beds on hard substrata in deep (> 60 m) habitats in the Main and Northwestern Hawaiian Islands (Huisman et al. 2007). In some areas off west Maui, these beds were found mixed with finger-like projections of the shallow mesophotic coral *Montipora capitata* (Spalding 2012). Little is known about its ecology or lifespan, although endemic reef fish were observed using these mesophotic beds for habitat in the Northwestern Hawaiian Islands (R. Kosaki, pers. comm.). The fine mesh was often intertwined with detritus and occasionally contained finely branched red algal epiphytes.


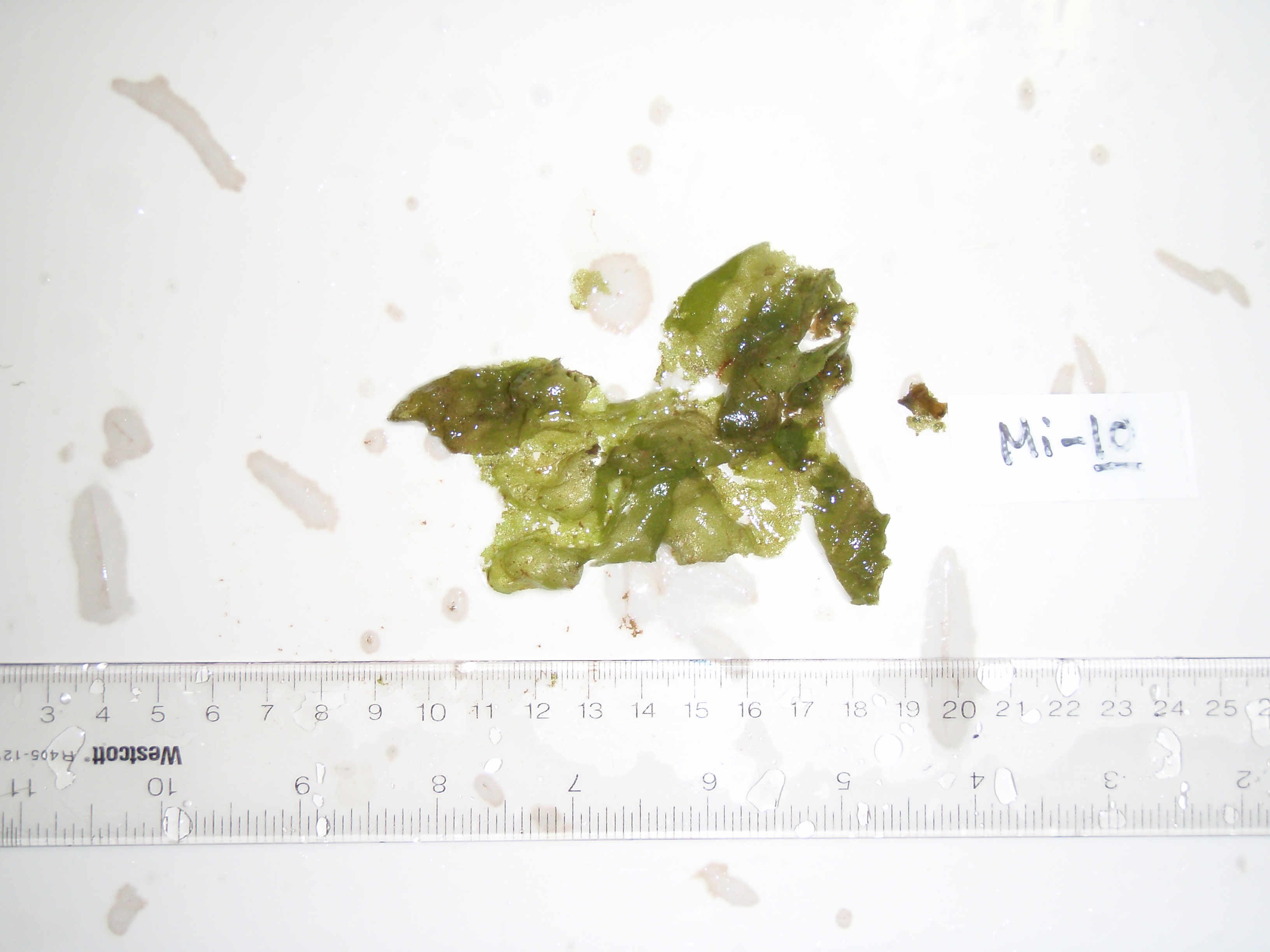


***Halymenia* sp.**

This species of *Halymenia* is not consistent morphologically with any species currently described in Hawai‘i, and it is likely a new species or new record. The genus *Halymenia* belongs in the red algal (Rhodophyta) order Halymeniales. Each specimen formed a large, single, smooth blade with a medulla of loosely intertwined filaments and was attached to hard substrata by a single, discoid holdfast. Individuals were frequently found as scattered individuals on hard substrata from 85 - 90 m depths, and were often associated with blades of green algae belonging to the genera *Ulva* and *Umbraulva*. The surface of the blade was slick and gelatinous, and did not host any epiphytes or encrusting organisms.


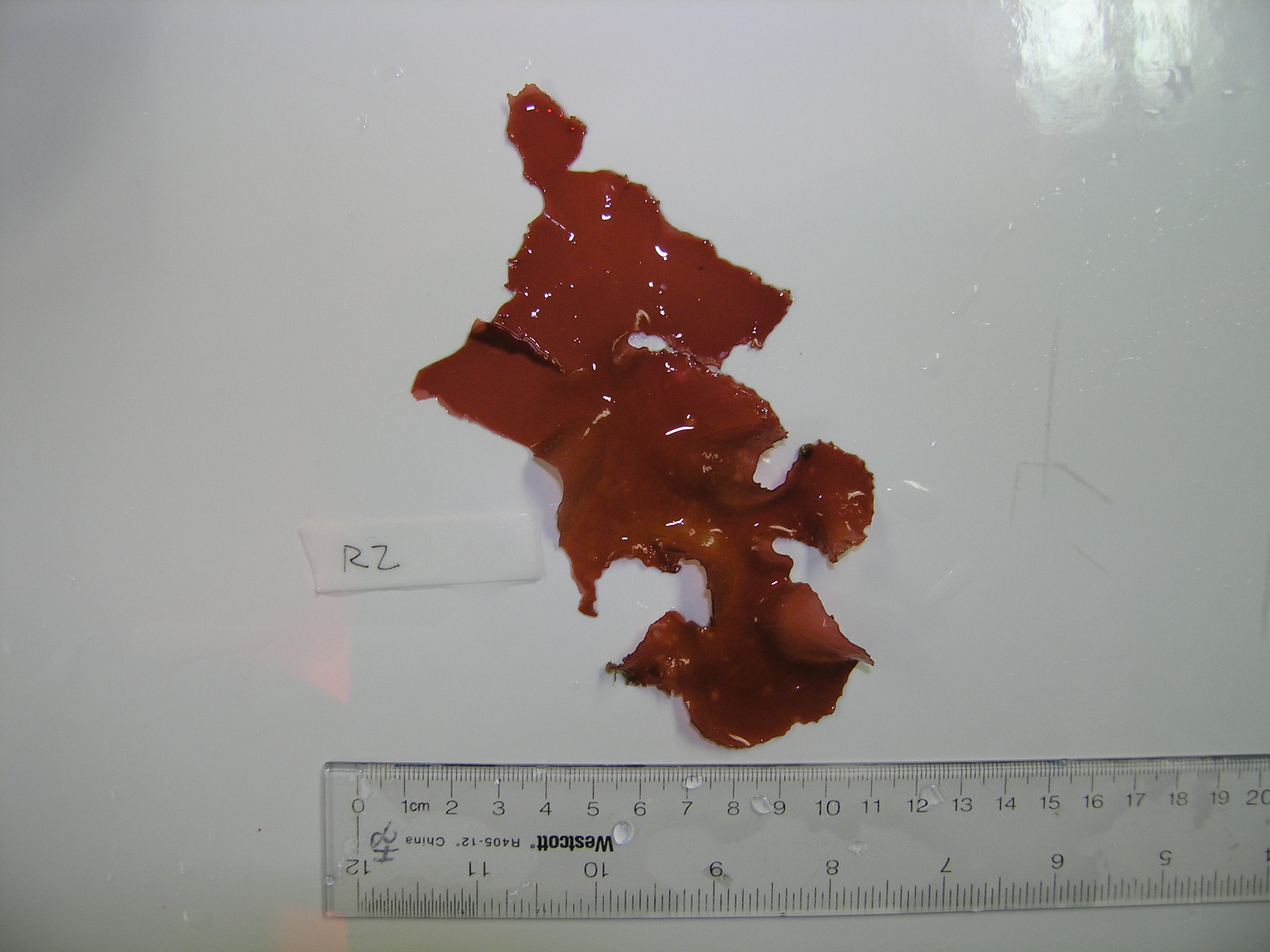


***Distromium* sp.**

*Distromium* sp. is a brown alga (Phaeophyceae) in the order Dictyotales composed of a kidney-shaped blade with only two cell layers. Ongoing molecular analyses suggest this genus is composed of three new species (Spalding et al. 2015). *Distromium* was the most commonly encountered brown alga in Hawai‘i’s mesophotic zone, with populations found in a range of habitats, such as with *Leptoseris* spp. and branching *Montipora capitata* coral reefs, in sand, and on carbonate substrate at mesophotic depths (44 - 160 m; Spalding 2012). The blades often grew epiphytically over other algae and coral, and often hosted the red algal epiphyte *Ditria reptans* on the distal surfaces or nongeniculate coralline algae on the basal portion of the blades. Algae within the Dictyotales are known to be highly chemically defended, and contain anti-herbivory, antifungal, cytotoxic, antibiotic, anti-inflammatory, insecticidal, or antiviral activities (Vieira et al. 2015). Although the genus *Distromium* has not been tested for these compounds, grazing marks were not observed on any thalli collected.


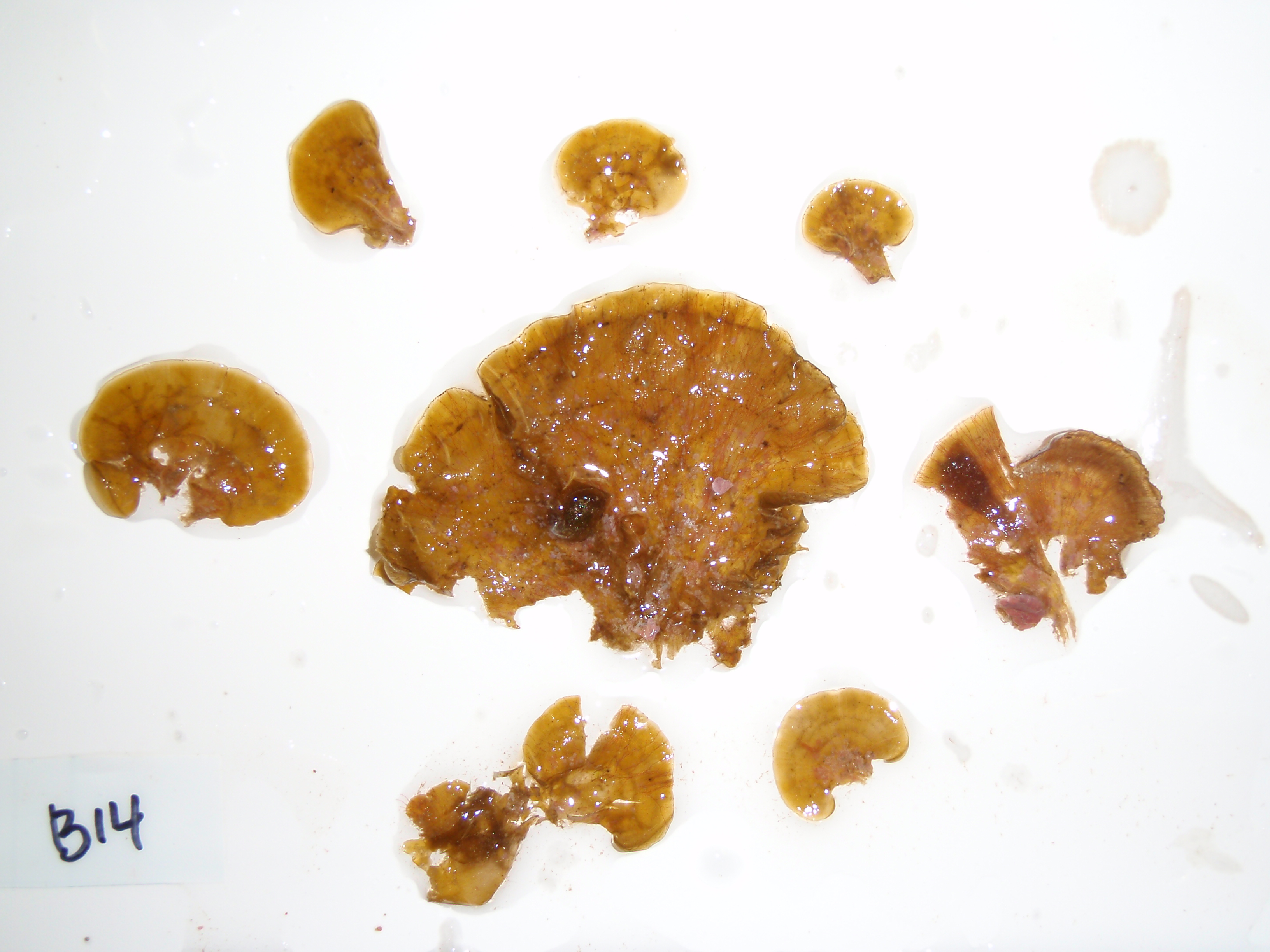


Literature Cited

Huisman JM, Abbott IA, Smith C. 2007. Hawaiian Reef Plants. Honolulu, Hawaii: University of Hawaii Sea Grant College, 264 pp.

Spalding H. 2012. Ecology of mesophotic macroalgae and *Halimeda kanaloana* meadows in the Main Hawaiian Islands. Honolulu, Hawaii: University of Hawaii, 199 pp.

Spalding HL, Conklin K, Tsuda RT, Sherwood AR. 2015. Diphering *Distromium* diversity from the deep. Phycological Society of America Annual Meeting, Philadelphia, PA, Aug 8 – 13, p 77.

Vieira C, Gaubert J, De Clerck O, Payri C, Culioli G, Thomas OP. 2015. Biological activities associated to the chemodiversity of the brown algae belonging to genus Lobophora (Dictyotales, Phaeophyceae). *Phytochemistry Reviews*: 1-17.
